# Immediate induction of varicosities by transverse compression but not uniaxial stretch in axon mechanosensation

**DOI:** 10.1186/s40478-022-01309-8

**Published:** 2022-01-24

**Authors:** Chao Sun, Lin Qi, Yang Cheng, Yi Zhao, Chen Gu

**Affiliations:** 1 Department of Biological Chemistry and Pharmacology, The Ohio State University, 182 Rightmire Hall, 1060 Carmack Road, Columbus, OH 43210 USA; 2 Department of Biomedical Engineering, The Ohio State University, Columbus, OH 43210 USA; 3 MCDB Graduate Program, The Ohio State University, Columbus, OH 43210 USA; 4 Department of Nutritional Sciences and Toxicology, University of California Berkeley, Berkeley, 94720 USA; 5 School of Optics and Photonics, Beijing Institute of Technology, Haidian District, No.5 Yard, Zhong Guan Cun South Street, Beijing, 100081 China

**Keywords:** Axonal varicosity, Mild traumatic brain injury (mTBI), Diffuse axonal injury (DAI), Transverse compression, Uniaxial stretch, Concussion, Neural plasticity, Microtubule

## Abstract

Uniaxial stretch is believed to drive diffuse axonal injury (DAI) in mild traumatic brain injury (mTBI). Axonal varicosities are enlarged structures along axonal shafts and represent a hallmark feature of DAI. Here we report that axonal varicosities initiate in vivo immediately after head impact and are mainly induced by transverse compression but not uniaxial stretch. Vertical and lateral impacts to the mouse head induced axonal varicosities in distinct brain regions before any changes of microglial markers. Varicosities preferentially formed along axons perpendicular to impact direction. In cultured neurons, whereas 50% uniaxial strain was needed to rapidly induce axonal varicosities in a nanowrinkled stretch assay, physiologically-relevant transverse compression effectively induced axonal varicosities in a fluid puffing assay and can generate large but nonuniform deformation simulated by finite element analysis. Therefore, impact strength and direction may determine the threshold and spatial pattern of axonal varicosity initiation, respectively, partially resulting from intrinsic properties of axon mechanosensation.

**Supplementary Information:**

The online version contains supplementary material available at 10.1186/s40478-022-01309-8.

## Introduction

Traumatic brain injury (TBI), a worldwide health problem, contributes to about a third of all injury-related deaths in the United States, while most of the 1.7 million TBIs that occur each year belong to mild TBI (mTBI) or concussion [1]. Among the mTBIs, those showing long-term deficits (~ 225,000) approximate to the combined number of patients diagnosed with breast cancer, multiple sclerosis or spinal cord injury each year [2]. However, the prognosis of long-term deficits among mTBIs remains extremely poor, in part due to the gap of knowledge regarding the primary injury that often involves a combination of different mechanical forces to the brain tissue, such as tension, compression, torsion and shear. Diffuse axonal injury (DAI) is the most common neuropathology in mTBI, in which uniaxial stretch is believed to play a central role. It has been postulated that uniaxial stretch induces microtubule breakage to disrupt axonal transport of various organelles and other cargos, causing axonal dysfunction and hence classical symptoms of decreased processing speed and memory impairment [3–7]. Whereas DAI has been extensively described using animal models and postmortem human tissues, mechanistic insights into axonal injury by uniaxial stretch have been provided by elegantly designed in vitro assays using primary neuron cultures combined with compartment chamber, stretchable membrane and/or magnetic tweezers [3, 8–11]. Despite progress, our understanding of mTBI primary injury remains highly limited, in contrast to more extensive studies of mTBI secondary injury.

Mechanosensation underlying the senses of touch, hearing and balance, and pain, has been well studied, but little is known about how central nervous system (CNS) neurons convert mechanical stimuli into changes in morphology and function. One possible mechanism underlying CNS neuron mechanosensation involves axonal varicosities, enlarged structures along axonal shafts [12, 13]. The geometric irregularity alone in axonal varicosities can profoundly affect axonal conduction and synaptic transmission [13, 14]. Our recent studies showed that fluid mechanical pressure through puffing rapidly (≤ 5 s) and reversibly (≥ 20 min half recovery) induced axonal varicosity formation in cultured CNS neurons [12]. Using Thy1-YFP transgenic mice with a subset of projection neurons expressing YFP, we further visualized axonal varicosity formation immediately after the second impact that took place 24 h after the first impact in a repeated closed-skull impact model (rcTBI) [12]. Consistent with our findings, several groups independently reported that axonal varicosities were observed from 3 h to 3 days after one impact in the brain of Thy1-YFP transgenic mice in different closed-skull mTBI models [15–17], as well as in an open-skull TBI model combined with immunostaining for amyloid precursor protein [18]. However, it remains unknown whether axonal varicosities can be induced in vivo immediately but not hours after one mechanical impact, just like rapid axonal varicosity formation in cultured neurons induced by fluid puffing shown in our recent study [12].

Many in vivo and in vitro model systems have been developed to study various aspects of TBI [19, 20], but cross-system studies are still very limited, hindering a better understanding of the core mechanism underlying CNS mechanosensation and injury. In the present study, by comparing two mTBI mouse models, we discovered that mechanical impact indeed induced axonal varicosity formation in mouse brains immediately after mechanical impact, representing the earliest subcellular event known in mTBI. Our results indicate that whereas impact strength sets the threshold of axonal varicosity formation, impact site and direction likely determine the spatial pattern of axonal varicosity initiation in the brain. Axonal varicosity induction preferentially formed along axons perpendicular to the impact direction, which is consistent with our in vitro results that transverse compression but not uniaxial stretch efficiently induced axonal varicosities using two biomechanical assays with primary neuron culture. Taken together, our results suggest that transverse compression may play a predominant role in mechanical stress-induced axonal varicosity formation, contributing to its spatial pattern in the brain during mTBI primary injury.

## Materials and methods

### Repetitive closed-skull TBI (rcTBI) model

The detailed procedure was previously described [12, 21]. In brief, male C57BL/6 J and Thy1-YFP-H transgenic mice (8 weeks old; Stock # 003,782 from the Jackson Laboratory) anesthetized with 5% isoflurane and placed in a stereotaxic frame with rounded Kopf head holders (David Kopf Instruments, Tujunga, CA, USA). Temperature was controlled at 37 °C using a feedback temperature controller (Cell Microcontrols, Norfolk, VA). Isoflurane was delivered by nose cone at 2% in air. The heads were shaved and prepped with Betadine. A midline skin incision was made and the skull was exposed. A rubber tip (Precision Associates, Inc., Minneapolis, MN, USA) was mounted on an electromagnetic stereotaxic impact device. The rubber tip was 9 mm in diameter and the rubber had a spring constant of 3.01 N/mm. The tip was fully extended and lowered at a 20-degree angle until the vertex touched the skull at 1.8 mm caudal to bregma and 3.0 mm left of midline. This was confirmed with a hand lens in all cases. The tip was then retracted automatically. The stereotaxic device was moved down by 3.3 mm, and the electromagnetic device was triggered, driving the tip 3.3 mm into the exposed skull at 5.0 m/s with a dwell time of 100 ms. The deformation of the rubber tip spread the impact force over the skull. There were < 3% skull fractures and no immediate fatalities after these injuries. Mice with skull fractures were killed and were not used in any experiments and mice with hemorrhages were excluded from the analyses. After impact, the skin was sutured and the mice were allowed to recover from anesthesia on a warming pad and then returned to their home cages. After 24 ± 1 h, a second identical closed-skull TBI procedure was performed, and then the mice were immediately perfused and fixed. For sham injuries, the same procedure was performed except that the impact device was discharged in the air; the handling of the mice and duration of anesthesia were the same for rcTBI and sham procedures.

### Closed-head impact model of engineered rotational acceleration (CHIMERA)

We adopted the CHIMERA system and performed the model as previously described [22, 23]. In brief, the system included an accumulator air tank, pressure regulator, digital pressure gauge, two-way solenoid valve, trigger button and a 50 g free-floating chrome-coated steel piston. We adjusted the pressure regulator to reach 5.36 psi shown in digital pressure gauge, which enabled reliable delivery of piston velocity (~ 6.0 m/s) and hence 0.9 Joule (0.9 J) impact energy. All animal handling procedures in the current study were approved by the Ohio State University Institutional Animal Care and Use Committee (IACUC) and conformed to the United States National Institutes of Health Guide for the Care and Use of Laboratory Animals. C57BL/6 J and Thy1-YFP-H transgenic mice (8–12 weeks old; from the Jackson Laboratory) were used. We did not detect any difference between male and female mice in axonal varicosity induction, so their results have been combined in this study. Mice were anaesthetized with isoflurane (induction: 4%, maintenance: 1.5%) in oxygen (0.9 L/min), which stopped right before impact. Lubricating eye ointment was applied to prevent corneal drying. Meloxicam (1 mg/kg) and saline (1 mL/100 g body weight) were administered by subcutaneous injections for pain control and hydration, respectively. The piston strikes the vertex of the head covering a 5 mm area at the midpoint between bregma and lambda. Impacted mice with regained breathing and heartbeat (usually < 15 s after impact) proceeded to cardiac perfusion immediately (from impact to cardiac perfusion < 2 min). Sham mice underwent all of these procedures, except for the impact. Approximately 3% of mice did not regain breathing after impact, and were thus euthanized and excluded from our analysis.

### Brain tissue fixation, sectioning, and immunostaining

The procedures of cardiac perfusion, fixation, sectioning, staining and imaging were described in our previous papers [24–27]. In brief, all mice were deeply anesthetized with Euthasol (Virbac; 100 mg/kg) and perfused transcardially with 20 ml of PBS followed by 20 ml of a 4% formaldehyde/PBS solution. Brains were removed and post-fixed for 1 h in 4% formaldehyde/PBS solution, and then were cut into 3-mm blocks using an acrylic brain matrix (Braintree Scientific, Braintree, MA, USA) and cryoprotected in a 30% sucrose/PBS solution for > 24 h. All parts from the same brain was arranged into one block, embedded in optimal cutting temperature (OCT) media (Sakura Finetek USA, Inc., Torrance, CA, USA), and stored at − 80 °C until sectioning. The tissue blocks were cut with a Microm HM550 cryostat (Thermo Scientific, Waltham, MA, USA) and the 40-µm sections were collected on Superfrost Plus microscope slides (FisherScientific, Pittsburgh, PA, USA) for storage at − 20 °C. After immunostaining, all sections were coverslipped using tris-buffered Fluoro-Gel mounting media (Electron Microscopy Sciences, Hatfield, PA, USA).

### Antibodies and immunofluorescence staining

Antibodies used in this study include rabbit polyclonal anti-neurofilament 200 antibody (Cat. #: N41421; Sigma, St. Louis, MO, USA), rabbit polyclonal anti-Iba1 antibody (Wako Pure Chemical Industries, Ltd., Osaka, Japan), rat monoclonal anti-CD68 antibody (Cat. #: MCA1957GA; Bio-Rad; Hercules, CA, USA), Cy3- and Cy5-conjugated secondary antibodies (Jackson ImmunoResearch Laboratories, West Grove, PA, USA). All antibodies were used in a 1:200 dilution. The nuclear dye Hoechst 33,342 and Fluorescein-conjugated Wisteria Floribunda Agglutinin (WFA) for labeling perineuronal nets were purchased from Invitrogen (Cat. #: H3570; Carlsbad, CA, USA) and Vector Laboratories (Cat. #: FL-1351–2; Burlingame, CA, USA), respectively. The procedure of immunofluorescence staining was previously described [26–28]. Sections were permeabilized in 1% Triton X/PBS for 1 h at room temperature (RT) and then blocked with 2.5% normal donkey or goat serum for 1 h at RT. Sections were then incubated overnight at 4 °C with the primary antibodies in blocking buffer. Twenty-four h later, sections were rinsed for 5 min seven times, incubated with the appropriate secondary antibodies in blocking buffer for 3 h at RT, counterstained with Hoechst 33,342 (1:2500) and/or WFA (1:500) for 10 min, and again rinsed for 5 min seven times. Stained slides were coverslipped with tris-buffered Fluoro-Gel mounting media (Electron Microscopy Sciences, Hatfield, PA, USA). Multiple staining rounds were carried out with at least one slide from each experimental group.

### Conventional fluorescent microscopy and spinning-disc confocal microscopy

Fluorescence microscopy and image analyses were carried out as described in previous publications from our laboratory [12, 26, 28–32]. Low magnification images were captured using a Spot CCD camera RT slider (Diagnostics Instruments, Sterling Heights, MI, USA) on a Zeiss Axiophot upright microscope with a 20X/0.50 Plan Apo objective and saved as 12-bit TIFF files. Exposure times were adjusted to ensure that pixel intensity in targeted tissue samples were below saturation, and kept constant across all experimental conditions for each of the specific fluorophores utilized within each round. Representative high magnification images were captured with an Andor Revolution WD spinning disk confocal system (Oxford Instruments, Abingdon-on-Thames, UK) based on a Nikon TiE inverted microscope using a 60X CFI Plan Apo VC water immersion objective with a numerical aperture of 1.40. Z-stack images (8-bit TIFF files) were taken for each region of interest at ~ 0.25 µm steps and flat images were generated using a maximum intensity projection. An YFP + axon with varicosities was defined as having the beads-on-a-string morphology (> 1 varicosity in every 20 μm length) and bead’s diameter > 200% the diameter of its adjacent shafts. All continuous YFP + axonal segments that were longer than 50 μm in length in an image stack (20X) were included to give rise to the number of total axons. For each brain region of each mouse, we captured 1–2 representative and non-overlapping image stacks in addition to random 2D images for quantification. We focused on the following brain regions, corpus callosum (CC) (Bregma between − 1.82 and − 2.30 mm), external capsule (EC) and cortical layer VI (Bregma between + 1.98 and + 1.78 mm), cortical layers I–V (Bregma between + 1.98 and + 1.78 mm), and hippocampus (Bregma between + 1.98 and + 1.78 mm). Closed-skull impacts, staining of brain slides and fluorescent microscopy were carried out in a blinded fashion.

### Mouse cortical neuron culture systems and transfection

Cortical neuron culture was prepared from mouse pups at postnatal days 1–3 (P1-P3) using the same procedure as previously described [29, 33]. Rat tail collagen and poly-D-Lysine were used to coat glass coverslips for neuron culture. In briefly, 2 d after neuron plating, 1 μM cytosine arabinose (Sigma-Aldrich, St Louis, MO, USA) was added to neuronal culture media to inhibit glial growth for the subsequent 2 d, then replaced with normal neuronal culture media. Culture media were replenished twice a week by replacing half volume. For transient transfection, neurons in culture at 5–7 DIV were incubated in Opti-MEM containing 0.8 μg of cDNA plasmid and 1.5 μl of Lipofectamine2000 (Invitrogen, Carlsbad, CA, USA) for 30 min at 37 °C.

### The nanowrinkled stretchable device and uniaxial stretching assay

An array of microscale rectangular cell loading membranes was fabricated using soft lithography. Linear nanoscale wrinkled structures are patterned on the top surface of the membranes and align to the longitudinal axis of the membranes. The expansion degree of loading membranes was calibrated with the air pressure. Loading membranes were coated with the same procedure as glass coverslips. Mouse cortical neurons were seeded on the membranes and their processes were often partially aligned due to the topographic cues provided by the nanowrinkles. Different air pressures provided by an air pump were used to induce different levels of uniaxial strain, which was further confirmed with the light microscope. In engineering, mechanical strain represents the relative displacement between particles in the body, which can be expressed by a tensor which 6 independent strain components (three normal strains and three shear strains). For the uniaxial stretching assay in this paper, the normal strain along the stretching direction can be expressed by ΔL/L, where L is the original length of the subject and ΔL is the change in length along the stretching direction upon loading.

### Fluid micromechanical pressure provided by local puffing

The fluid puffing system was previously described [12, 34]. To provide local fluid puffing, the glass pipette (tip diameter ~ 50 μm) was connected to a syringe via tubing filled with 20 ml Hank’s buffer and elevated at 190 mm above the tip of the pipette. The vertical distance between the pipette tip and cultured neurons was set at 0.4 mm. The formation of axonal swelling was considered a varicosity when it was ≥ 200% width of its adjacent axonal shafts. The onset time is defined as the time for an axonal segment to reach 10 varicosities per 100 μm length during puffing. Of note, under normal conditions without puffing, axon diameters are not perfectly uniform with the presence of a low level of varicosities, similar to the in vivo situation. Thus, the baseline of varicosity density along axons is not absolute zero.

### Live-cell timelapse imaging and transmission electron microscopy (TEM)

Neurons growing on 25 mm coverslips or loading membranes were incubated with Hank’s buffer at room temperature 15 min before imaging experiments. The time-lapse imaging setup was built on a Nikon TE2000 inverted microscope. Images were captured with a CCD camera Coolsnap HQ (Photometrics, Tucson, AZ, USA) through yellow fluorescent protein (YFP) or other filter sets with 1 s exposure time. The filters were changed through filter wheels controlled via Lambda 10–3 (Sutter Instrument, Novato, CA, USA) by the MetaMorph software (Molecular Devices). Time-lapse imaging was performed with indicated intervals for hundreds of frames. This procedure was described in our previous papers [24, 35]. For TEM, cultured cortical neurons with or without puffing were fixed with 4% paraformaldehyde and 3% glutaraldehyde in 0.1 M phosphate buffer, pH 7.4. Embedding, sectioning, and imaging with TEM were done in the Campus Microscopy and Imaging facility at The Ohio State University as described previously [12, 31].

### Finite element modeling

The deformation and mechanical strain of the axon was estimated using finite element analysis (COMSOL Multiphysics 5.5) (Analyzing the Mechanical Behavior of Cells for Biological Applications | COMSOL Blog by Fallqvist 2018) [36]. The axon is assumed a homogenous and hyperelastic material. The properties of cytoplasm are used since cytoplasm occupies the majority of the cell body and the entire axon. The axon is modeled as a semi-cylindrical body fixed on the bottom substrate. The fluid puffing is simplified as a uniform pressure applied to the half surface of the semi-cylinder. A 2D model is established with the puffing pressure ranges from 0 to 250 Pa. The first principal strain was plotted on the deformed axon (side view). We use first principal strain to represent the strain magnitude. The principal strains refer to the strains that are normal to the planes where the shear strains are zero. The first principal strain is the algebraically largest principal strain.

### Statistical analysis

Results were presented as the mean ± SEM. Two-tailed Student’s t-test was used for comparisons between two groups. One-way ANOVA followed by Dunnett’s test was used for comparing two or more groups to one control group. (*) *p* < 0.05 and (**) *p* < 0.01 or 0.001 were considered statistically significant.

#### Results

### Immediate induction of axonal varicosities in closed-skull mTBI models

**Fig. 1:**
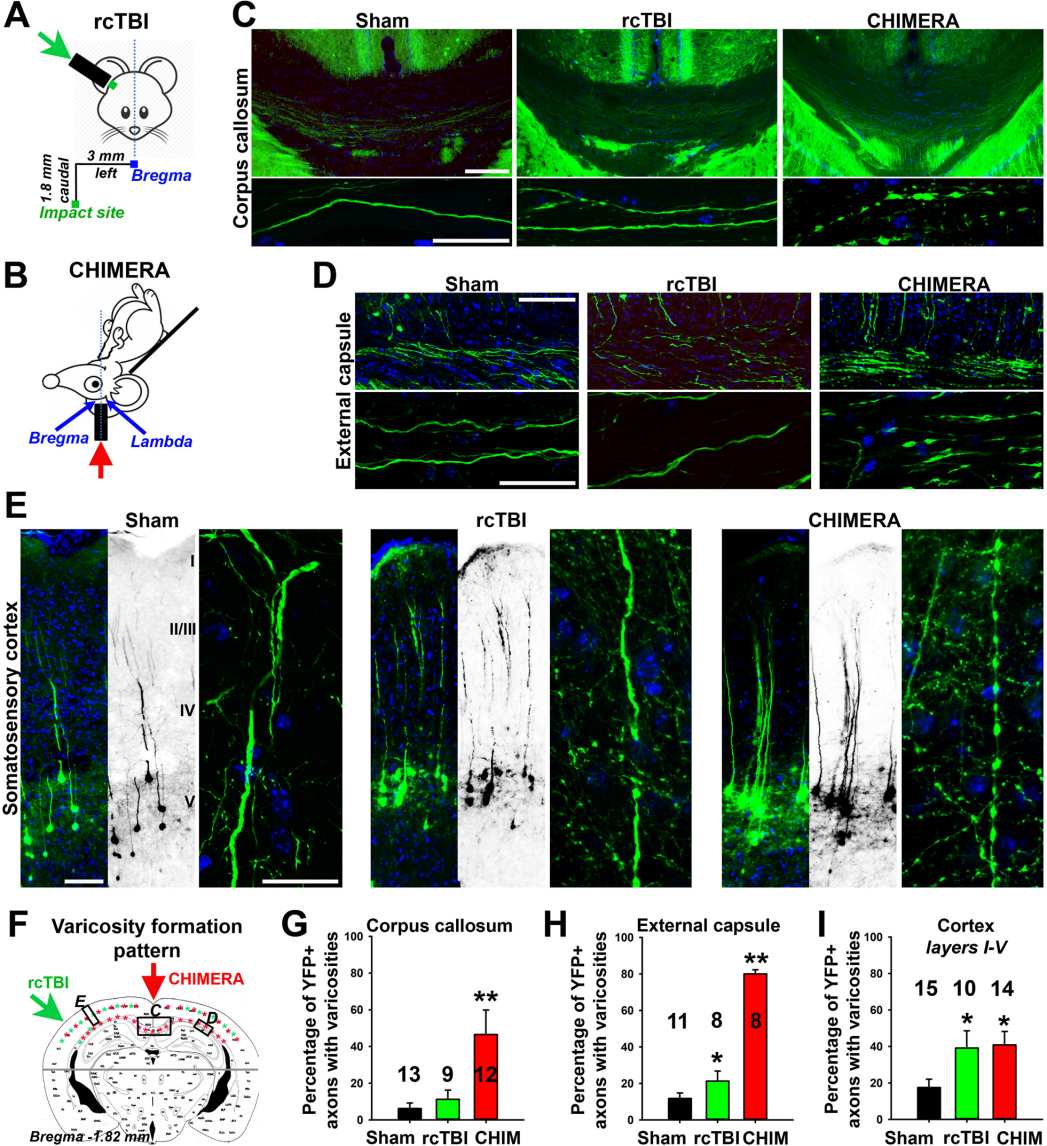
Different spatial patterns of axonal varicosity initiation in rcTBI and CHIMERA. Experiments were performed with Thy1-YFP transgenic mice (2–4 months old, male). YFP fluorescence and Hoechst (a nuclear dye) are shown in green and blue, respectively. **A** Impact diagram of rcTBI (perfusion and fixation immediately after the 2^nd^ impact). Green arrow indicates the impact direction. Impact site: 3 mm left and 1.8 mm caudal of bregma. **B** Impact diagram of CHIMERA (perfusion and fixation immediately after one 0.9-J impact). Red arrow indicated the impact direction. Impact site: the bregma. **C** CHIMERA but not rcTBI or sham induced clear axonal varicosities in corpus callosum (CC) at bregma − 2.06 mm. High magnification confocal images are provided in lower panels. **D** Extensive formation of axonal varicosities in external capsule (EC) of CHIMERA but not sham or rcTBI. High magnification confocal images are provided in lower panels. **E** Axonal varicosity induction by both rcTBI and CHIMERA in the cortex. High magnification confocal images are provided on the right for each condition. **F** Diagram of the spatial pattern of axonal varicosity induction in rcTBI (green asterisks) and CHIMERA (red asterisks). The brain regions above the gray line were the focus of our analysis. Boxed regions were analyzed in **C**, **D** and **E**. **G**, Summary for the percentage of YFP + axons containing varicosities in CC. Image numbers are provided in the figure. **H** Summary for the percentage of YFP + axons containing varicosities in in EC. Image numbers are provided in the figure. **I** Summary for the percentage of YFP + axons containing varicosities in cortical layers I-V. Image numbers are provided in the figure. Mouse numbers: 8 sham, 5 rcTBI and 6 CHIMERA. Unpaired t-test: *, *p* < 0.05; **, p < 0.001. Scale bars, 250 μm in low mag images and 20 μm in high mag images in **C-E**

To determine whether axonal varicosities form in vivo immediately but not hours after a mechanical impact, we adopted the Closed-Head Impact Model of Engineered Rotational Acceleration (CHIMERA), which closely mimics concussion [22, 23]. Both rcTBI and CHIMERA are closed-skull mouse models for mTBI. Their major differences are impact position and head movement post impact, besides the impact number (Fig. 1A,B). In rcTBI, lateral impact took place on one side of the head (3 mm left of the midline and 1.8 mm caudal of bregma). The mouse head was immobilized and impacted twice with 24 h (h) interval. Mice were immediately perfused and fixed after the 2nd impact. In CHIMERA (0.9 J), mice received only one vertical impact on the top of the head (at the midpoint between bregma and lambda) and the head moved after impact. Thus, there were additional linear and rotational loadings from acceleration and deceleration of head movement in CHIMERA. We used Thy1-YFP transgenic mice, in which a subset of projection neurons express YFP, and perfused the mice immediately after CHIMERA impact. YFP fluorescence allowed clear visualization of morphological changes in axons and dendrites of these neurons, including axonal varicosities. A substantial amount of axonal varicosities formed in the corpus callosum (CC) and external capsule (EC) in CHIMERA, whereas no significant increase in CC and a relatively small but significant increase in EC in terms of axonal varicosity formation were found in rcTBI (Fig. 1C,D,F–H and Additional file 2-5). Actually, the highest level of CHIMERA-induced axonal varicosities was found in the EC (Fig. 1G-I). In the cerebral cortex, rcTBI induced axonal varicosities in a multifocal fashion in the superficial cortical layers with a bias around the impacted side, whereas CHIMERA induced axonal varicosity formation throughout all cortical layers (Fig. 1E,I and Additional file 6, 7). Thus, different closed-skull impacts can induce distinct spatial patterns (partially overlap) of axonal varicosities in the brain. More importantly, since there are normally less than 2 min between impact and cardiac perfusion, our results show that mechanical impact can indeed very rapidly, or immediately, induce axonal varicosity formation in vivo, representing the earliest subcellular event that is known in mTBI primary injury.

In CHIMERA, we tested two additional levels of impact energy, 0.5 J and 0.7 J. Both impact energies caused significant increase of axonal varicosities and 0.5 J was most likely the threshold energy, consistent with earlier threshold studies by examining mouse behaviors and staining intensities of endogenous markers [23]. There was a clear correlation between impact strength and abundance (& size) of induced axonal varicosities, but the spatial pattern of varicosity formation largely remained the same, mainly in EC, CC and cortical layers (data not shown). For consistency, we used 0.9 J CHIMERA throughout this paper. Taken together, our results of rcTBI and CHIMERA have further suggested that whereas impact strength sets the threshold, impact direction/site may be a key determinant of brain-region specificity in mTBI-induced axonal varicosity formation at the initial stage of primary injury.

### Varicosities are preferentially initiated along the axons perpendicular to impact direction

The spatial pattern of axonal varicosity formation and injury may contribute to predictions of injury-induced neurological symptoms and long-term outcomes. However, it remains unknown what is the impact variable(s) associated with the spatial pattern of primary injury. Potential variables include the impact strength, number, site and direction. In rcTBI, no significant formation of axonal varicosities was observed in CC, in stark contrast with massive formation of axonal varicosities in CC in CHIMERA (Fig. 1C). CC axons are highly parallel to each other, but their orientations to the impact direction of rcTBI and CHIMERA are drastically different. Thus, these CC axons, as well as axons in many other brain regions, received different amounts and types of mechanical forces in the two mTBI models with different impact directions.

**Fig. 2:**
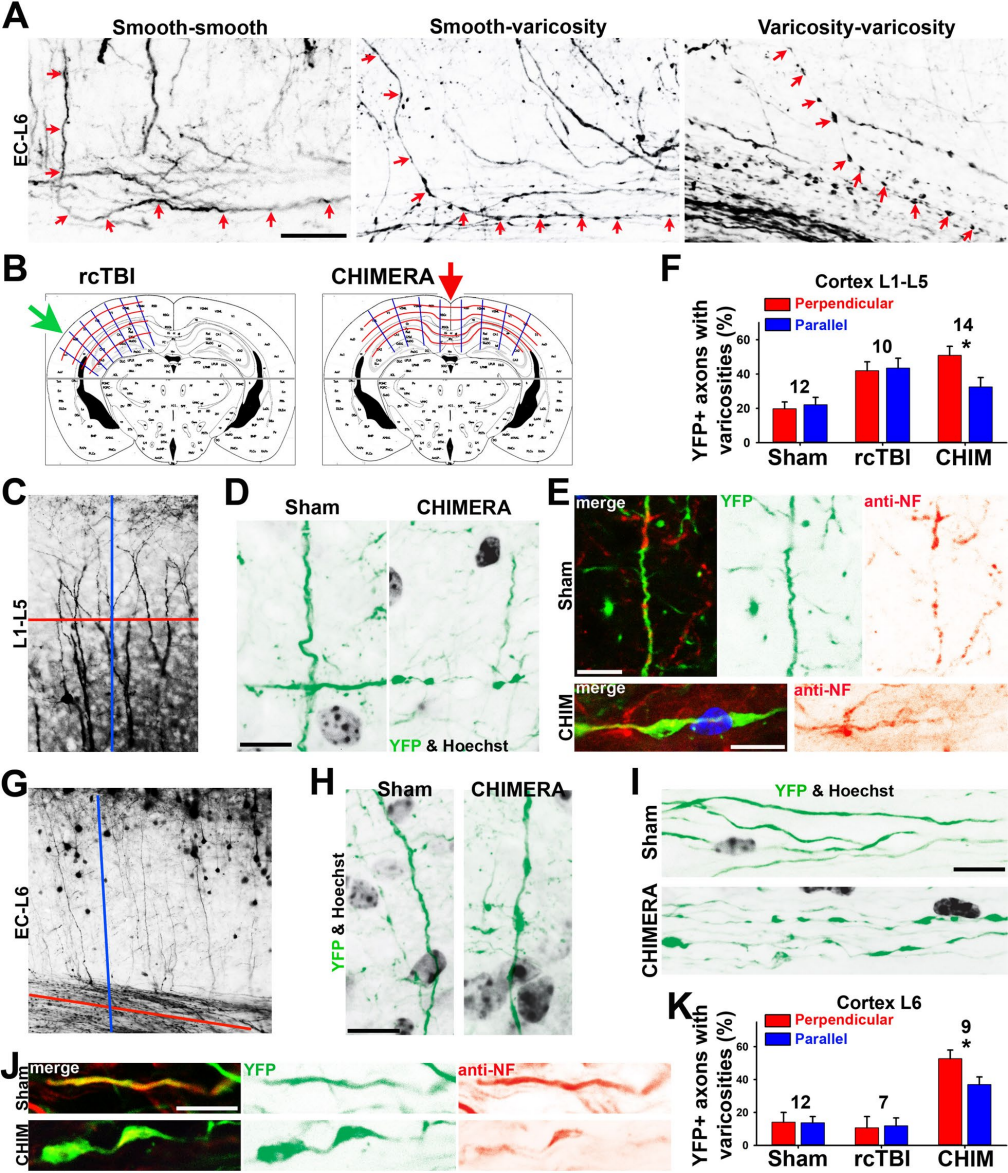
Correlation between varicosity formation and axon orientation to impact direction. **A** Axons (red arrows) from L6 to EC containing no varicosity (left, sham), varicosities in the EC portion (middle, CHIMERA) or varicosities in both sections (right, CHIMERA). YFP signals are inverted in gray scale. **B** Orientations parallel (blue) or perpendicular to (red) the impact direction in rcTBI (left) and CHIMERA (right). The brain regions above the gray line were the focus of our analysis. **C** YFP + dendrites of layer V pyramidal neurons and YFP + axons in cortical layers 1–5 (L1–L5). YFP signals are inverted. **D** High magnification confocal images showing that axons (L1-L5) perpendicular to the impact direction had more varicosities in CHIMERA. **E** YFP + axons (green) partially colocalizing with NF (red) in cortical layer 3 in sham (top) and axons containing varicosities with NF staining in CHIMERA (bottom). **F** Summary of percentage of YFP + axons (perpendicular (red) or parallel (blue) to the impact direction in the same image) with varicosities in L1-L5 in mouse brains of sham, rcTBI and CHIMERA. Image numbers are provided above the bars. Mouse numbers: 8 sham, 5 rcTBI and 6 CHIMERA. **G** Axons of YFP + layer 5 pyramidal neurons near external capsule (EC). YFP signals are inverted. In the L6-EC region of CHIMERA mice, different levels of varicosities in axons parallel (**H**) or perpendicular (**I**) to the impact direction. **J** YFP + axons (green) partially colocalizing with NF (red) in EC in sham (top) and axons containing varicosities with NF staining in CHIMERA (bottom). **K** Summary of percentage of YFP + axons (perpendicular (red) or parallel (blue) to the impact direction in the same image) with varicosities in L6 in mouse brains of sham, rcTBI and CHIMERA. Image numbers are provided above the bars. Mouse numbers: 8 sham, 5 rcTBI and 6 CHIMERA. Unpaired t-test: *, p < 0.05. Scale bars, 25 μm in **A** and 10 μm in **D**, **E**, **H-J**

In this Thy1-YFP transgenic mouse line, EC had the highest percentage of axons that developed varicosities in CHIMERA (Fig. 1G–I). Some axons in cortical layer VI can be observed in the same focal plane to make 90 degree turn and merge into the EC. Within these axons, we observed the following three types in sham and CHIMERA mice, (1) smooth segment in layer VI and smooth segment in EC (mostly in sham), (2) smooth segment in layer VI and varicosities in axonal segment in the EC in CHIMERA, (3) varicosities in both axonal segments in layer VI and EC in CHIMERA (Fig. 2A). Thus, there were axons with varicosities only formed within a segment that was perpendicular to the impact direction. This is consistent with our recent in vitro results that puffing-induced axonal varicosity formation is a highly localized event and only occurs within the puffing region [12]. This in vivo result suggests that mechanical stress-induced axonal varicosity formation is a highly localized event, at least at the early stage, and those axons perpendicular to impact direction are more likely to contain varicosities.

To further determine whether axon orientation under specific impact direction correlates with the level of axonal varicosity formation, we thoroughly analyzed different brain regions in CHIMERA and rcTBI (Fig. 2B). CHIMERA caused transverse compression onto CC axons and most EC axons, while rcTBI only directly caused transverse compression onto ipsilateral EC axons, but not CC axons. However, it became more complicated in the cortex due to the axons with mixed orientations (Fig. 2C). YFP + axons in cortical layers I-V confirmed with staining for neurofilaments (NFs; an axonal marker) can be perpendicular or parallel (or somewhere in between) to the impact direction, and a significant higher percentage of perpendicular axons contained varicosities in CHIMERA, but not in sham or rcTBI (Fig. 2C–F). Of note, NF staining signals in YFP + axons were present but not highly enriched in axonal varicosities (Fig. 2E).

Cortical layer VI (gray matter) is adjacent to EC (white matter) at the junction of gray and white matter. Whereas EC contained the highest percentage of axons with varicosities in CHIMERA (Fig. 1G-I), the level of induced-axonal varicosities in the cortical layer VI was relatively lower, and comparable to the level in layers I-V (Fig. 2G–K). Thus, axons in the cortical layer VI adjacent to the gray-white-matter interface did not contain more varicosities after CHIMERA impact. There were varicosities formed along axons parallel to the impact direction, but there were significantly more axons perpendicular to the impact direction that contained varicosities (Fig. 2H,I). In rcTBI, although some axonal varicosities can be observed in EC, there was no clear increase of axonal varicosity formation in the layer VI (Figs. 1H and 2K). Similar to layers I–V, significant higher percentage of axons perpendicular to than those parallel to the impact direction in the layer VI contained varicosities in CHIMERA, but not in sham or rcTBI (Fig. 2K). Those axons in the EC also sometimes contained NF staining, and with a low or no enrichment in axonal varicosities (Fig. 2J). Therefore, NF is a widely used marker for axons, but unlikely useful for revealing axonal varicosities in mTBI.

### Axonal varicosity formation precedes adjacent microglial activation after closed-skull impact

**Fig. 3:**
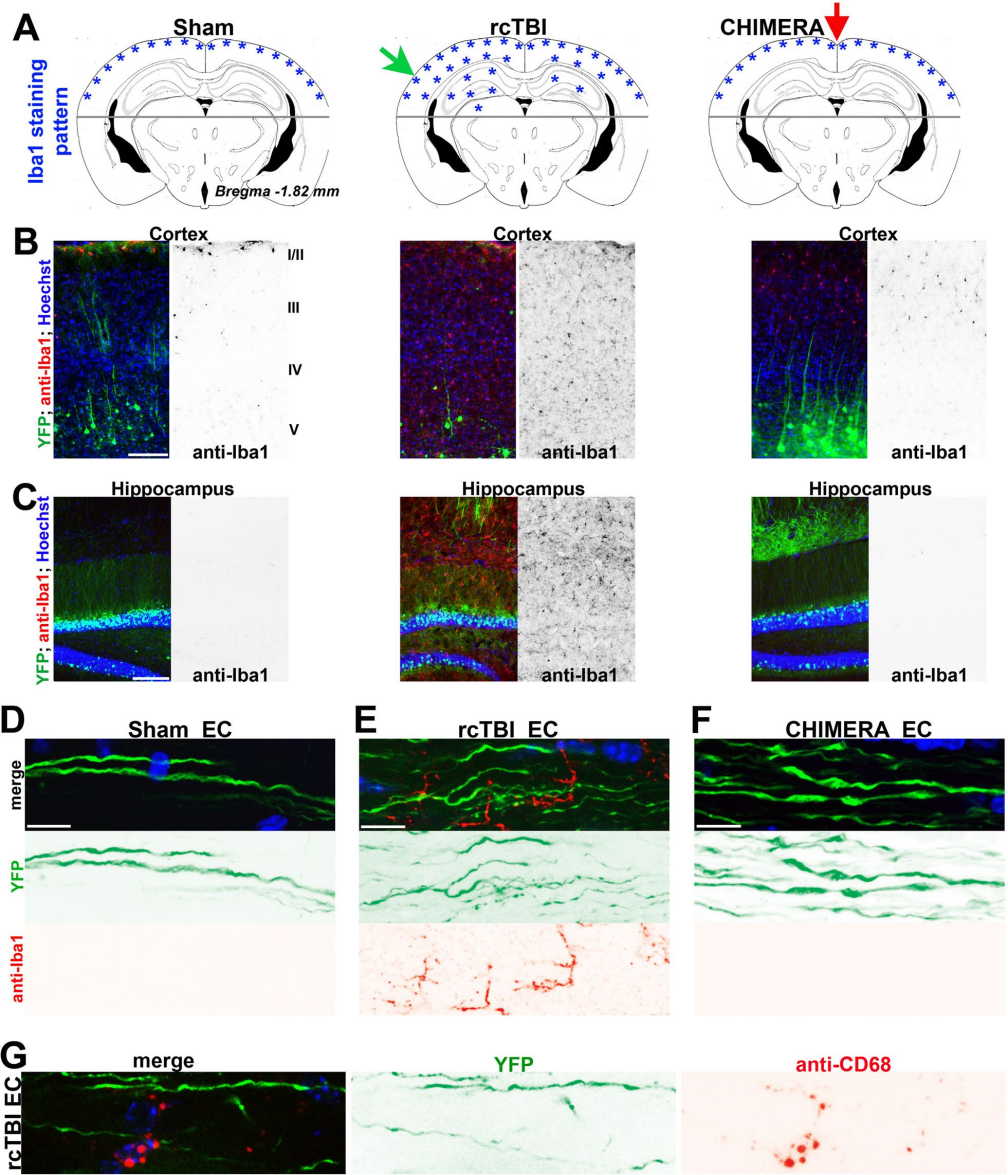
Delayed induction of Iba1 upregulation in rcTBI and CHIMERA. **A** Distribution pattern diagrams of significant Iba1 staining in the brains from sham, rcTBI and CHIMERA (0 h). Green and red arrows, impact sites in rcTBI and CHIMERA, respectively. The position of brain section diagram, Bregma − 1.82 mm. The brain regions above the gray line were the focus of our analysis. **B** Significantly upregulated Iba1 staining signals (red in the merged images) throughout all cortical layers of Thy1-YFP mice (YFP in green) in rcTBI (middle), but not in sham (left) and CHIMERA (right). **C** Strong Iba1 staining signals in the hippocampus of Thy1-YFP mice in rcTBI (middle), but not in sham (left) and CHIMERA (right). **D** Smooth YFP + axons in Sham EC with no significant Iba1 staining signal. **E** Smooth and varicosity-containing YFP + axons in rcTBI EC with significant Iba1 staining signals. **F** YFP + axonal varicosities in CHIMERA EC with no adjacent Iba1 signal. **G** An example of YFP + smooth axons (green, with no varicosity) with nearby CD68 + microglia (red) in EC from rcTBI brain. Scale bars, 250 μm in **B**, **C** and 15 μm in **D-G**

Due to relatively low levels of axonal varicosity formation in CC and EC in rcTBI compared to CHIMERA, we wondered whether this might result from an overall lower level of brain injury in rcTBI, compared to that in CHIMERA. As a key pathological feature of mTBI, microglial activation is commonly indicated by upregulation of ionized calcium-binding adaptor protein-1 (Iba1) and morphological transition from the ramified resting state into the hypertrophied bushy phenotype [37]. Reactive microglia appeared mainly proinflammatory at the early stage of the mTBI injury [38]. Seven days after the first impact in rcTBI, early studies showed microglial activation in cortex, hippocampus, EC and CC with some bias on the impact side [21]. On the other hand, extensive microglial activation was seen 2 days after impact in multiple white matter regions in CHIMERA [22, 23]. In the present study, we used immunofluorescent imaging and found that in sham mouse brain, the overall expression level of Iba1 was quite low throughout various brain regions including the hippocampus, EC, CC and cortical layers II-VI, except the cortical layers I-II where Iba1 staining was relatedly higher (Fig. 3A–C). Moreover, these Iba1-positive cells displayed ramified morphology, consistent with that of resting microglia. Right after the second impact in rcTBI, extensive increases of Iba1-positve cell density and hypertrophied morphology were present in those brain regions (Fig. 3A–C and Fig. S1A,B), highly consistent with the early report [21]. Importantly, the increases occurred on both sides of the brain, though being biased on the impacted side (Fig. S1A). Immediately after CHIMERA (a single impact), we did not observe any significant change of Iba1 staining, compared to sham, consistent with early results [23]. Overall, rcTBI brains appeared to clearly have higher level of neuroinflammation than CHIMERA brains right after a single impact (Fig. 3A–C), indicating significant brain injury, despite the lower level of axonal varicosity formation in CC and EC in rcTBI.

To further determine whether there was any correlation between axonal varicosity formation and adjacent microglial activation, we performed confocal microscopy on brain slides stained for two different microglial markers. In rcTBI, clear increase of Iba1 + cell density was observed in EC, where smooth axons without any clear varicosities were often found nearby (Fig. 3D, E). In contrast, axonal varicosities in EC, CC and cortical layer VI induced by CHIMERA did not colocalize with Iba1 + cells or significant Iba1 signals nearby (Fig. 3F). Of note, Iba1 is a pan-microglial marker. To verify the increase of neuroinflammation, we performed the staining against CD68, which a transmembrane glycoprotein highly expressed by cells in the monocyte linage and often used as a marker for proinflammatory reactive microglia. The CD68 staining results were consistent with those from Iba1 staining. For instance, CD68 + microglia were often found near smooth axons in EC in rcTBI, but not in Sham or CHIMERA (Fig. 3G). Taken together, despite relatively less axonal varicosity formation in CC and EC, rcTBI induced a significantly high level of microglial activation throughout many brain regions. Therefore, initial formation of axonal varicosities appears to precede and not to require local microglia activation. A caveat here is that since only a subset of axons expressed YFP in this transgenic mouse line, it remains to be determined whether these features can be applicable for all axons.

To determine whether the different patterns of microglial activation resulted from single impact in CHIMERA versus two impacts in rcTBI, we performed repeated CHIMERA with 24 h interval similar to rcTBI. Indeed, there was a significant increase of Iba1 staining and deramified microglia overall in repeated CHIMERA in the CC, EC, cortical layers I-V and cerebellar cortex, compared to single CHIMERA, whereas Iba1 staining density remained relatively low in the hippocampus in repeated CHIMERA, in contrast to rcTBI (Fig. S2). Actually, throughout later time points in single CHIMERA (up to 1 month), significant microglial activation by Iba1 density was present in CC, EC and cortex, but not in the hippocampus, which is consistent with earlier reports [22, 23, 39]. For Iba1 staining of the resting microglia, it is important to note that the DAB staining used in those studies is more sensitive, whereas immunofluorescence staining used here is suitable for double labeling and intensity quantification. Thus, lateral impact but not vertical impact appeared to more effectively induce microglia activation in the hippocampus in the mouse brain, consistent with our hypothesis that the injury pattern in the brain correlates with impact site/direction. Taken together, our results suggest that each suprathreshold impact with particular site and direction induces a distinct pattern of axonal varicosities followed by a delayed and partially overlapping pattern of microglial activation in mTBI.

### Axonal varicosities can be rapidly induced in vitro by uniaxial stretch with 50% or more strain

Currently still lacking is a clear understanding of the transmission of mechanical loads from the scale of tissues to axons. A simple head impact can generate heterogeneous mechanical stresses in different brain regions (e.g. with some regions compressed but others stretched). In both mTBI models described above, especially CHIMERA, axon orientation highly correlated with the induction level of axonal varicosities—axons perpendicular to the impact direction contained more varicosities compared to the axons parallel to the impact direction (Fig. 2). One possibility is that depending on their location and orientation, some axons are compressed while others were stretched during mechanical impact. Among various types of mechanical forces, uniaxial stretch was postulated as the major force to induce DAI [3–7]. In fact, uniaxial stretch was the most frequently used way of load application among all TBI in vitro models published in the past decade or so [20].

**Fig. 4:**
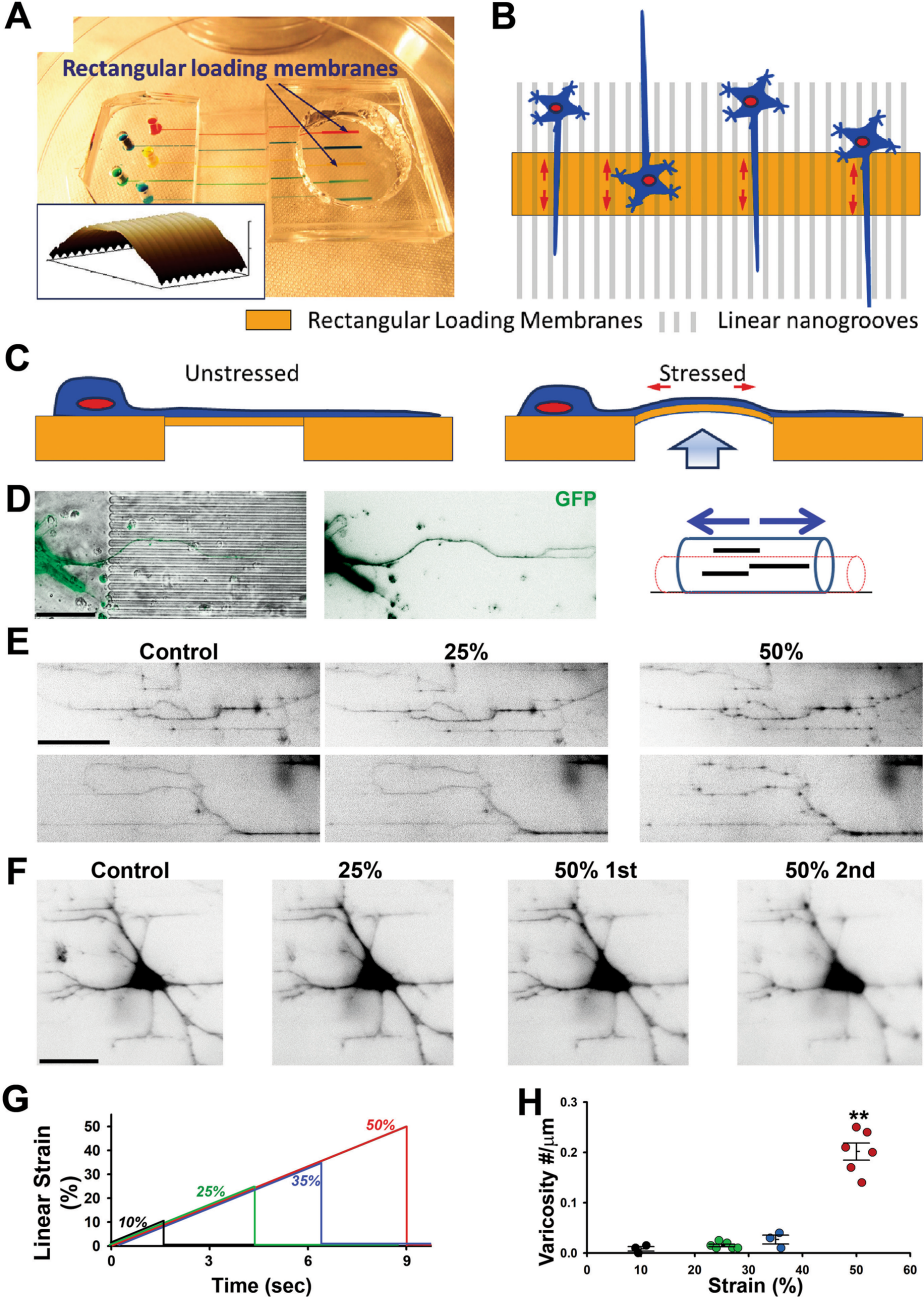
Induction of axonal varicosities of cultured cortical neurons by uniaxial stretch in a nanowrinkled stretch device. **A** A fabricated cell loading device with four rectangular membranes. The inset shows the atomic force microscopic image of the nanowrinkled surface upon membrane deformation. **B** The top-view schematic of neurons growing on the top of stretchable surface. **C** The side-view schematic of cell loading under control (unstressed and left) and stressed (right) conditions. **D** A cultured cortical neuron expressing YFP (green) with its axon growing into stretchable membrane region. Axon uniaxial stretch diagram, right. **E** Axonal varicosities induced by 50% strain (right), but not by 25% strain (middle). YFP signals are inverted. **F** Varicosities formed in dendrites after being stretched twice with 50% strain. **G** Summary of the relationship between target linear strain and time. **H** Summary of axonal varicosity formation under different target strains. One-way ANOVA followed by Dunnett’s test: **, *p* < 0.01. Scale bars, 35 μm in **D**, **F** and 15 μm in **E**

To determine whether uniaxial stretch can effectively induce axonal varicosity formation in cortical neurons, we developed a nanowrinkled stretch assay to study cultured cortical neurons. An array of microscale rectangular cell loading membranes was fabricated using soft lithography (Fig. 4A–C). Linear nanoscale wrinkled structures were patterned on the top surface of the membranes and aligned to the longitudinal axis of the membranes. Mouse cortical neurons were seeded on the membranes and cultured for up to 2 weeks. Their processes were often partially aligned due to the topographic cues provided by the nanowrinkles (Fig. 4D). In-plane strain was applied to the neurons via the membranes and the strain direction was along the nanowrinkles. For the axons growing strictly along the nanowrinkles, uniaxial stretch was the only type of mechanical force that they received (Fig. 4D). The relationship between the air pressure and nanowrinkled membrane strain was calibrated. The cortical neurons transfected with YFP were imaged with a conventional fluorescent microscope before stretching. The nanowrinkled and stretchable membrane moved out of the focal plane of the microscope during stretching (2–9 s depending on the target strain, or a similar strain rate ~ 0.06 s^−1^), and quickly returned back to the original focal plane after reaching the target strain. Then, the neurons were imaged again post stretching.

We tested the effects of a variety of target strains on both axonal and dendritic morphology of cultured cortical neurons. 10–35% strain did not cause any clear morphological changes in axons, including varicosity induction, and the minimal strain to reliably induce axonal varicosities was 50% (Fig. 4E–H and Fig. S3). Similar to our puffing results [12], dendrites were relatively more resistant to form varicosities compared to axons, but could develop varicosities in distal branches after repeated stretching with 50% strain (Fig. 4F). The relationship of linear strain and stretch time was plotted (Fig. 4G). Therefore, in our nanowrinkled stretch assay, the minimal strain to induce varicosity formation from axons or dendrites was around 50% (Fig. 4H). However, combining tagged magnetic resonance imaging (MRI) and digital image analysis, an early study provided a dense set of displacement measurements in the human brain during mild frontal skull impact and showed that the maximum principal strain was only around 5% [40]. A more recent computation modeling suggested that material heterogeneities at the gray-white interface could lead to a highly nonuniform distribution of stress in axons, reaching the maximal strain around 25% near the interface [41]. Similar maximal strain was reported from another computation modeling for multi-axial acceleration loading in human brain [42]. Therefore, our results from nanowrinkled stretch assay suggest that the maximal strain of uniaxial stretch in the brain during mTBI may be too small to induce axonal varicosities. Of note, axons are viscoelastic, so a caveat here is the strain rate. The strain rate is a key factor in uniaxial stretch-induced axonal injury and will be discussed in a later section.

### Physiologically-relevant transverse compression efficiently induces axonal varicosities

**Fig. 5:**
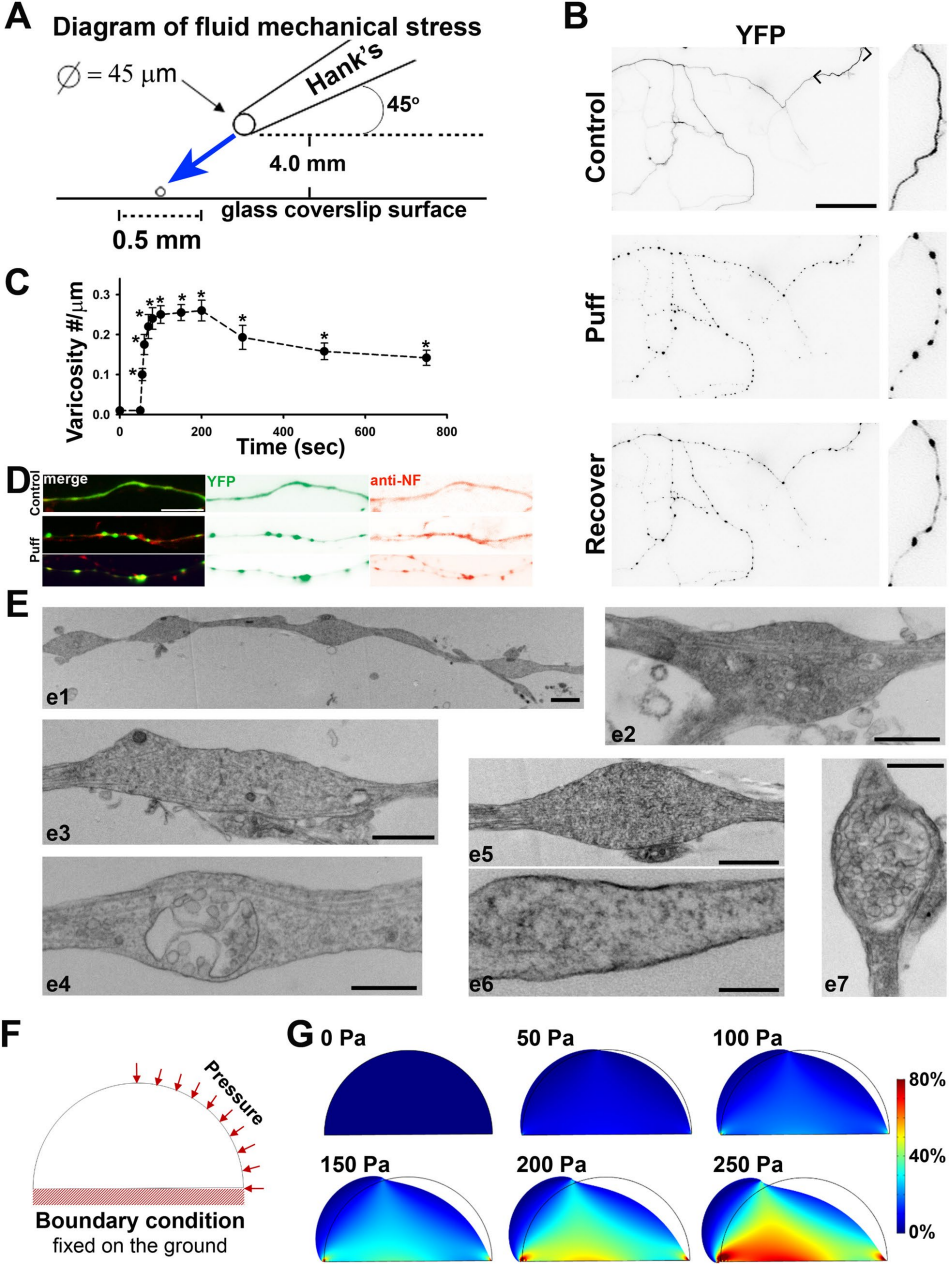
Transvers compression rapidly and reversibly induced axonal varicosity formation in cultured cortical neurons in the fluid puffing assay. **A** Diagram of fluid puffing set up, providing transverse compression. **B** Initiation and recovery of axonal varicosities in cultured cortical neurons by puffing. Cultured cortical neurons were transfected with YFP cDNA at 6 DIV and imaged at 8–10 DIV. YFP signals are inverted. **C** Summary of the density of axonal varicosities induced by 150-s puffing. Unpaired t-test: *p < 0.05. n = 7. **D** Colocalization of YFP (green) and NF staining (red) in control axons (top) and axons with varicosities post puffing (middle and bottom). **E** Heterogeniety of puffing-induced axonal varicosities revealed by TEM. e1, several varicosities along an axon; e2-e4, mitochondria and multivasicular body in axonal varicosities with several microtubule filaments; e5-e6, amorphies structures with no microtubule filaments in varicosities; e7, extensive and exclusive vesicular structure within the varicosity. **F** Diagram of the cross section of an axon with the loading and the boundary condition in the simulation. **G** The maximum component of the first principal strain under different pressures in the simulation with finite element analysis. The strain level is indicated by color. Scale bars, 30 μm in **B**, 15 μm in **D**, and 2 μm in **E**

Using fluid puffing assay, we recently showed that axonal varicosity formation induced by mechanical stress was unexpectedly rapid (≤ 5 s) and partially reversible (≥ 20 min for half recovery) in cultured hippocampal neurons [12]. This puffing system was modified based on a local drug perfusion system (Fig. 5A), in which the exact same solution was used for both the puffing pipette and the bath each time. Using this system, we tested multiple levels of puffing pressures and found that varicosity induction speed correlated with the pressure value [12]. Our micromeasurement showed that the minimal pressure that rapidly and reliably induced axonal varicosities was 0.25 ± 0.06 nN/µm^2^ (or 250 ± 60 Pa) onto cultured neurons at the center of puffing area, while the static pressure at the tip of puffing pipette was 190 mmH_2_O (~ 1.863 nN/μm^2^ or ~ 1863 Pa) [12, 34]. This pressure value onto the neurons is well within the estimated physiological range in the brain [12, 43]. Here, by using this system, we examined the effect of fluid puffing onto cultured mouse cortical neurons and found that axonal varicosity induction in a similar fashion to our previous in vitro results (Fig. 5B,C). Axonal varicosities were induced rapidly (~ 5 s) and could slowly and incompletely recover (Fig. 5C). We also stained for endogenous NF and found that NF was relatively enriched in some axonal varicosities but not in others (Fig. 5D). This moderate level of NF present in induced axonal varicosities is consistent with our in vivo staining results. To further determine the ultrastructure of induced axonal varicosities, we performed transmission electron microscopy (TEM). Our TEM results show that among puffing-induced axonal varicosities: ∼50% of them with a few MTs remaining, ∼30% without any visible microtubule filament, and ∼20% with extensive vesicular structures (some with mitochondria- and multivesicular body-like structures) (Fig. 5E). Taken together, transverse compression appeared to more effectively induce axonal varicosities, compared to uniaxial stretch.

How do we quantitatively compare the results from the nanowrinkled stretch and fluid puffing assays? The pressure and strain values carry different meaning. However, both types of mechanical forces, uniaxial stretch and transverse compression, induce local deformation in neurons, leading to neuronal mechanosensing and adaptive responses. Thus, to further understand why fluid puffing was more effective in axonal varicosity induction than uniaxial stretch, we performed a 2D finite element analysis for puffing induced local deformation, in order to convert pressure values into deformation or strain to allow comparison between the two biomechanical assays. The mechanical strain within the cells was estimated using COMSOL software bundle. For the sake of simplicity, the axon was assumed to have a semicircular cross-section and firmly bonded to the substrate. The axon was modelled as a hyperelastic body, with the shear modulus of 0.155 kPa and the bulk modulus of 1000 kPa, which were adopted from a relevant study [36]. A distributed pressure was loaded on the right half side (Fig. 5F), with the magnitude ranging from 0 to 250 Pa. The results showed that even a modest distributed pressure could induce considerably large, albeit nonuniform, strains, or local deformation. In particular, a 250 Pa pressure could generate 50% or more strain in more than half of the axonal area, and up to 80% strain in a small axonal area (Fig. 5G). Assuming the strain distribution was uniform in the nanowinkled stretch assay, the 50% strain (50% longer than the original length) would be identical across the entire axon (Fig. 4). Therefore, transverse compression with the physiological-relevant value (250 Pa) can cause nonuniformly distributed local deformation, which is at least on average comparable to, if not bigger than, the one induced by uniaxial stretch with 50% strain.

## Discussion

In the present study, using two different closed-skull impact mouse models, we have shown that mechanical impact can immediately induce axonal varicosity formation in mouse brains, representing the earliest subcellular event known in mTBI. The spatial pattern of initial formation of axonal varicosities in the brain appears to correlate with impact site/direction, which is partially explained by our in vitro results that transverse compression is more effective than uniaxial stretch in axonal varicosity induction. Our simulation has further indicated that transverse compression produces nonuniform but huge local deformation.

CHIMERA induced extensive formation of axonal varicosities in multiple brain regions, which happened immediately after impact (< 2 min between impact and cardiac perfusion) (Fig. 1). To our knowledge, this is the first report for such rapid induction. This in vivo result is highly consistent with our in vitro finding using cultured CNS neurons with the onset time ≤ 5 s [12]. Among published studies using closed- and open-skull models, the earliest observation of axonal varicosity formation so far was 3 h after impact [16, 44]. Moreover, our results confirmed extensive but delayed microglial activation in both mTBI models (Fig. 3), and further showed that axonal varicosities could initiate without nearby microglial activation revealed by markers (Iba1 and CD68), whereas microglial activation could happen adjacent to the axons without any varicosity (Fig. 3D–G). Thus, these results also suggest that microglial activation in mTBI may not require adjacent axonal varicosity formation. Whether and how activated microglia can promote the formation of axonal varicosities during the secondary injury remains to be determined. Moreover, it is possible that certain form of significant axonal injury does not involve varicosity formation or any other morphological change, and therefore cannot be detected by our imaging. Such form of axonal injury remains to be identified in future studies. Nonetheless, axonal varicosity formation is the earliest known subcellular event so far in neuronal injury in mTBI, and can become a valuable biomarker for studying the primary injury in mTBI.

Could different patterns of axonal varicosity formation in rcTBI and CHIMERA result from their difference in head movement post impact? Using the similar setup and procedure as our rcTBI model, a recent study showed that one impact onto the bregma (similar impact site and direction as our CHIMERA model) led to extensive axonal varicosity formation in the CC and EC [15]. A caveat here is that this study used Thy1-YFP-16 mouse line, in which slightly more neurons express YFP compared to Thy1-YFP-H mice used in our studies [15]. Nonetheless, a vertical impact like CHIMERA without head movement post impact like rcTBI caused a similar spatial pattern of axonal varicosities in the brain as CHIMERA but not rcTBI, suggesting that impact site and direction but not head movement post impact, play a key role here. Furthermore, using microglial markers, we found that rcTBI appeared to cause more wide-spread neuroinflammation across brain regions than CHIMERA, indicating that the impact strength of our rcTBI was adequate and not likely the cause of the difference (Fig. 3). Therefore, whether the head moves or not after impact appears not important in terms of the pattern of axonal varicosity formation. Of note, this notion does not argue against that acceleration and/or deceleration loading itself can be sufficient to cause brain injury. Taken together, whereas impact strength dictates the threshold, impact site and direction likely contribute to the spatial pattern of axonal varicosity formation.

Little is known regarding the relationship between the nature of impact and neurophysiological impairment. Our present study revealed an interesting correlation between axonal orientation related to impact direction and varicosity level in CHIMERA and rcTBI (Fig. 2). This may result from localized nature of axonal varicosity formation, as shown by our in vivo results (Fig. 2A) and in vitro data [12]. Our studies focused on an early time window after impact. Within this time period, axonal varicosity formation might not yet spread into other brain regions, maintaining a rather specific spatial pattern of axonal varicosities. However, it is important to note that although rcTBI’s effects on CC and EC axons were consistent with our conclusion, in the cortex rcTBI did not induce biased formation of varicosities along axons perpendicular to the impact direction (Fig. 2). This may partially result from several limitations in our analysis, including limited axon representation by YFP + ones, difficulty in group assignment of axons running neither parallel nor perpendicular to impact direction, indirect transverse compression by surrounding cells for axons parallel to impact direction. Currently, it is also technically challenging to analyze induced axonal varicosities in the hippocampus due to the high density of YFP + neurons and their processes. Limited representation of axon species in the present study can be addressed by follow up studies using transgenic mouse lines expressing fluorescent proteins in different types of neurons.

Axon swelling and pathology are commonly found in the deep gyri at the interface between the gray and white matter across all severities of TBI [4, 41]. Computation modeling showed that material heterogeneities lead to a highly uneven distribution of uniaxial strain in axons, with the highest level (~ 25%) in axonal regions from the interface to ~ 10 μm into the gray matter [41]. In contrast, our results from CHIMERA show that axonal varicosities were preferentially within the EC (white matter) at the EC-L6 interface (Fig. 2A). Furthermore, our results using nanowinkled stretch assay show that, to reliably and rapidly induce axonal varicosities, minimal 50% uniaxial strain was required (Fig. 4), in which the strain was evenly distributed in the axons growing along nanowrinkled grooves. This strain amplitude (50%) is not only the twice of the maximal strain in the gray-white matter interface predicted by computation [41], but also approximately one order of magnitude higher than the local strain measured by imaging [40]. Thus, our results question whether uniaxial stretch is the main mechanical force that drives DAI. However, an important caveat here is the rate of strain. It was reported that at slow strain rates, axons are able to withstand substantial strains, whereas axonal damage occurs at high strain rates for the same magnitude of strain [41]. The strain rates in our nanowrinkled stretch assay were relatively slow (0.05 to 0.06 s^−1^) and it took about 9 s to reach 50% strain (Fig. 4). However, stretch assay results with much faster strain rates and various amplitudes had been reported before from multiple groups [3, 9, 11]. Under those conditions, even after many repeated fast stretches, it took from 10 min to several hours for axons to develop clear varicosities [3, 9, 11]. It is possible that uniaxial stretch causes microinjuries of some subcellular structures, such as microtubules, before axons develop varicosities. Clearly, those previous results also indicated that uniaxial stretching is not an effective way to induce axonal varicosities, consistent with our results in the present study.

In stark contrast to uniaxial stretch, transverse compression by fluid puffing effectively induced axonal varicosities. The puffing pressure (0.25 ± 0.06 nN/μm^2^ or 250 ± 60 Pa) onto the neurons in our experimental setting does fall within the range of what previous investigators used in in vitro and in vivo studies. Using scanning force microscopy, the applied stress (0.27 ± 0.04 nN/µm^2^) altered exploratory behaviors of the leading edge of neurite processes in vitro in a neuronal cell line [45]. Using brain volumetric and physical data extracted from the literature, a recent study estimated 3.43 mmHg (~ 0.46 nN/μm^2^) in a normal human brain [12, 43]. Although it is still not feasible to accurately measure micromechanical pressures within a living brain, another study used fluorescent cell-sized oil microdroplets with defined mechanical properties to measure the maximal anisotropic stress generated by mesenchymal cells in living embryos at ∼1.6 ± 0.8 nN/µm^2^  [46]. Despite the small value of the puffing pressure, our simulation has showed that transverse compression can induce huge local strains that are nonuniformly distributed in part due to axon’s elastic properties, and a major portion of the axon is subjected to more than 50% strain under a 250-Pa pressure, leading to a pattern of local deformation at least comparable to that induced by 50% uniaxial strain (Fig. 5). Actually, this result is highly consistent with the notion that material heterogeneities contribute to large and nonuniform strains. This is because, for an axon, the material heterogeneity (e.g. axially aligned microtubules and plasma membranes) is intrinsically higher in the transverse direction than that in the longitudinal direction. Moreover, different types of mechanical forces (e.g. transverse compression versus uniaxial stretch) may preferentially regulate different mechanosensitive ion channels that are responsible for Ca^2+^ influx leading to axonal varicosity formation. Our recent studies indicated that transient receptor potential V4 (TRPV4) channel functioned as a major mechanosensitive ion channel in this process [12], and might be preferentially activated by transverse compression, which still needs to be validated in future experiments. Despite advantages for mechanistic studies, in vitro systems usually do not possess the microenvironment similar to the in vivo situation. In particularly, the two in vitro systems used in the present study do not contain myelin. How myelin regulates axonal varicosity formation induced by transverse compression and uniaxial stretch remains to be determined in future studies. Taken together, perhaps in both central and peripheral nervous systems, axons possess robust elastic property in the uniaxial direction and hence are resistant to stretch-induced injury, but are more sensitive to transverse compression-induced damage.

In summary, our findings lay a solid foundation for future mechanistic investigations of potentially distinct mechanosensitive signaling pathways for specific types of mechanical forces (e.g. transverse compression and uniaxial stretch) in the CNS. Moreover, our findings likely have translational or clinical implications in mTBI. For instance, frontal or vertical impact may preferentially damage the CC, whereas lateral impact may damage the hippocampus at the primary injury stage. However, mouse and human brains differ considerably. Whether the two impact directions correlate with behavioral and cognitive alterations related to CC and hippocampal (or any other brain regions) functions in mice and humans remains to be further investigated.

## Supplementary Information

**Additional file 1**. Supplemental figures and text.

**Additional file 2**. Video1: YFP+ axons in sham corpus callosum.

**Additional file 3**. Video2: YFP+ axons in CHIMERA corpus callosum.

**Additional file 4**. Video3: YFP+ axons in sham external capsule.

**Additional file 5**. Video4: YFP+ axons in CHIMERA external capsule.

**Additional file 6**. Video5: YFP+ axons in sham cortex.

**Additional file 7**. Video6: YFP+ axons in CHIMERA cortex.
