# Supplementary material for: Immediate induction of varicosities by transverse compression but not uniaxial stretch in axon mechanosensation
Source: Acta Neuropathol Commun. 2022 Jan 24;10:7. doi: 10.1186/s40478-022-01309-8 (PMC8785443; doi:10.1186/s40478-022-01309-8)
Supplement: Supplementary file 1 — Additional file 1. Supplemental figures and text. [file 40478_2022_1309_MOESM1_ESM.docx]

**Immediate Induction of Varicosities by Transverse Compression but Not Uniaxial Stretch in Axon Mechanosensation**

Chao Sun^1,3^, Qi Lin^2,4^, Yang Cheng^2,5^, Yi Zhao^2^, and Chen Gu^1,3^*

**Supplemental Information**

**
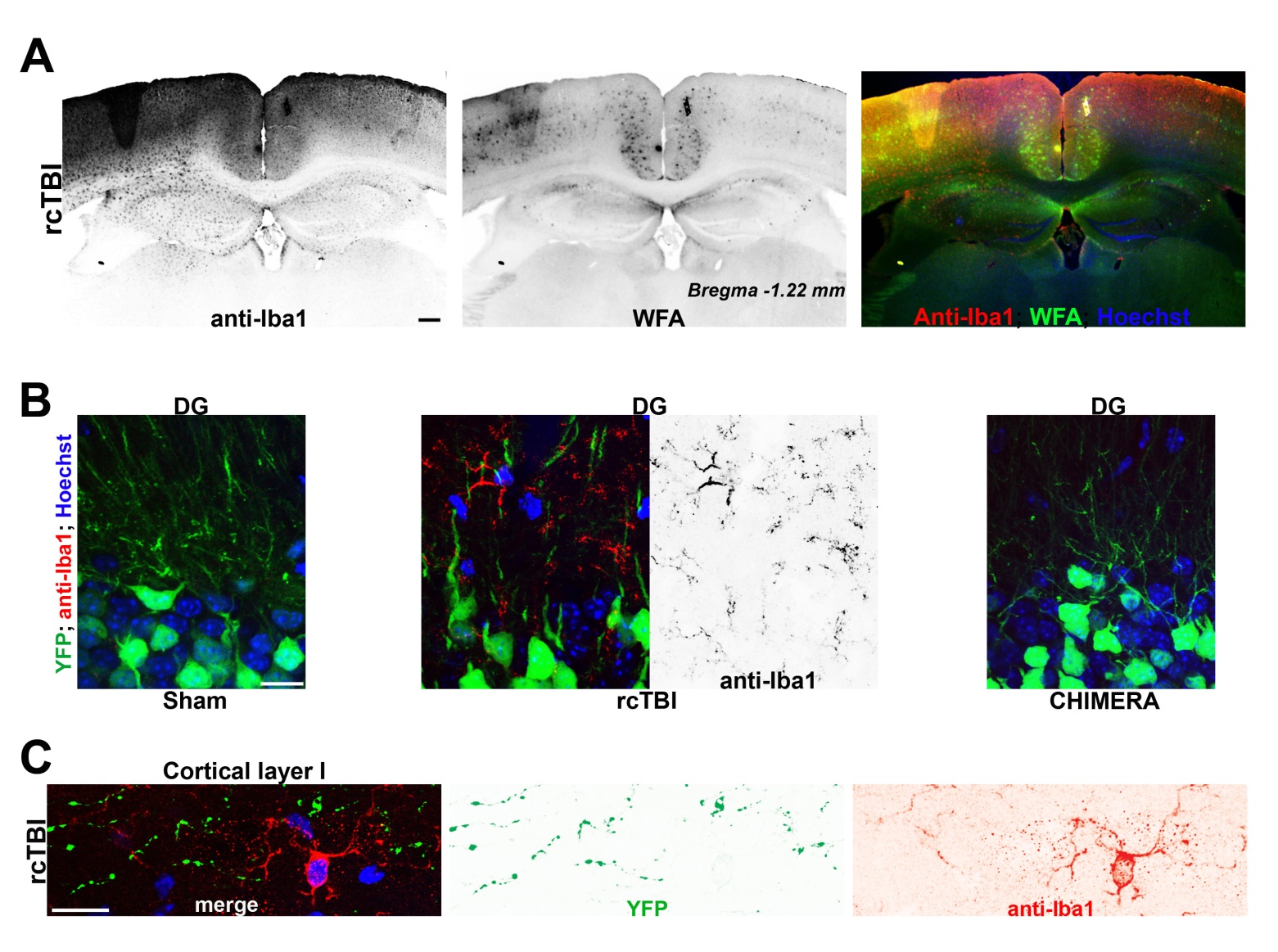
**

**Figure S1. Differential microglia activation in two mTBI mouse models.**

B6 WT and Thy1-YFP transgenic mice were used in the closed-skull impact models, rcTBI and CHIMERA.

**A**, The asymmetric distribution pattern of upregulated Iba1 staining signals in an rcTBI brain (WT C57B6/J mouse). The impact site is on the left. The position of coronal section, bregma -1.22 mm. Iba1 and WFA (Fluorescein-conjugated Wisteria Floribunda Agglutinin) staining signals were shown in inverted gray scale on the left and middle, respectively. In merged image on the right, Iba1 (red), WFA (green) and Hoechst (blue).

**B**, High magnification confocal images in the hippocampus dentate gyrus (DG) of Thy1-YFP mice show microglial activation in rcTBI (middle), but not in sham (left) and CHIMERA (right). In merged images, YFP in green, Iba1 staining signals in red and Hoechst in blue.

**C**, YFP+ axonal varicosities and adjacent Iba1 signals in the outer layer of cortex in rcTBI.

Scale bars, 800 μm in **A**, 40 μm in **B** and 15 μm in **C**.

**
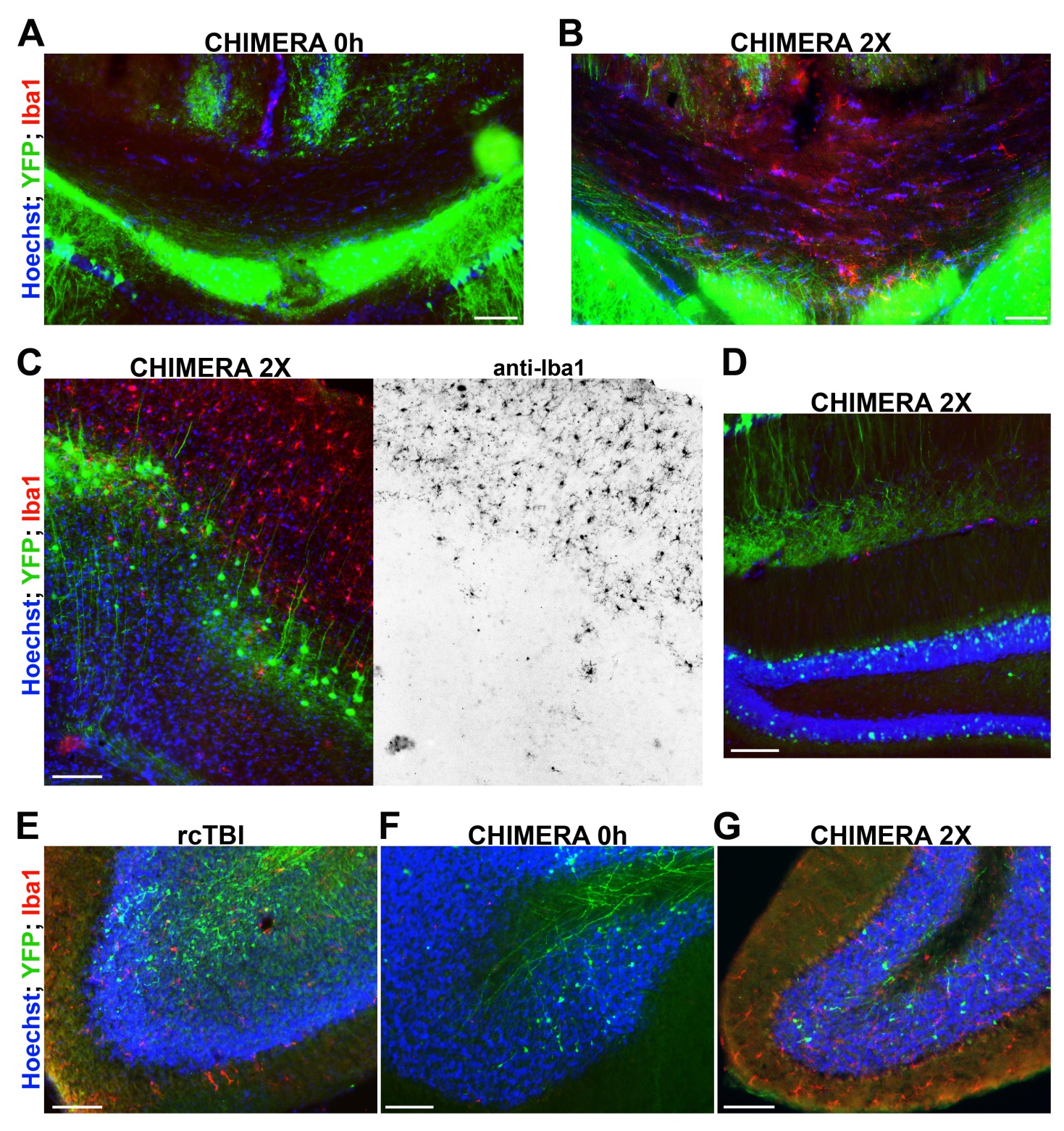
**

**Figure S2. Iba1 staining signals increased in repeated CHIMERA (2X) compared to a single CHIMERA (0h).**

Thy1-YFP transgenic mice were used here. Compared to a single CHIMERA (0h) (**A**), Iba1 signals (red) significantly increased in CC of Thy1-YFP transgenic mice that received repeated CHIMERA (2X) (**B**). YFP in green and Hoechst in blue.

**C**, Iba1 staining signals increased in the cortex of 2X CHIMERA.

**D**, There was still relatively low level of Iba1 staining signal in the hippocampus of 2X CHIMERA.

**E-G,** Significant increase of Iba1 staining signals in the cerebellar cortex in rcTBI and 2X CHIMERA but not in sham or CHIMERA (0h).

Scale bars, 500 μm in **A** and **B**, 250 μm in **C-G**.

**
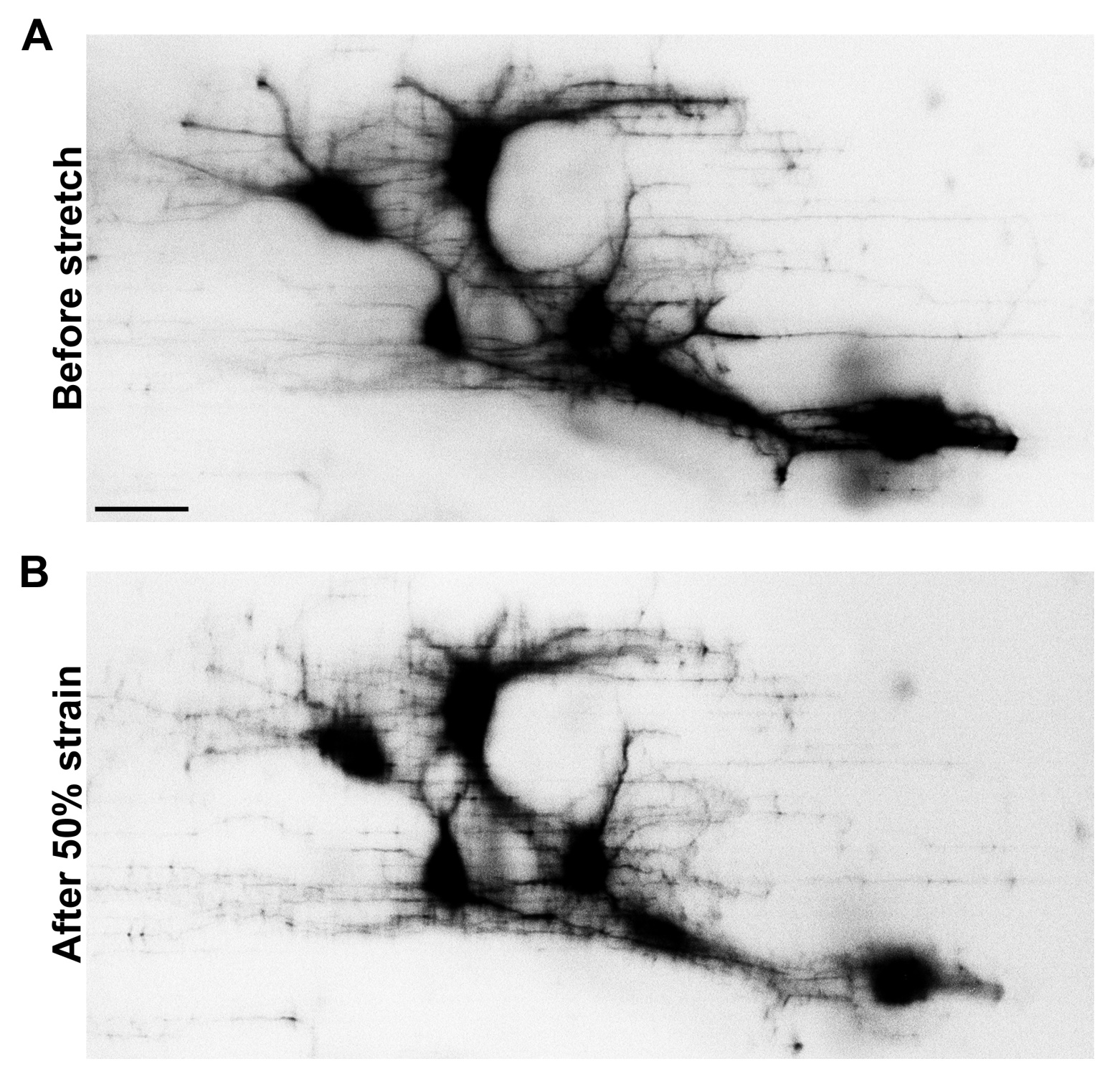
**

**Figure S3. Uniaxial stretch induced axonal varicosities with 50% strain in the nanowrinkled stretch device**

**A,** Six cortical neurons were cultured on the nanowrinkled and stretchable membrane at 12 DIV. The image was taken before stretching and fluorescence signals of transfected YFP were inverted.

**B**, The membrane with the neurons was stretched for 50%. The image was captured after the stretch when the membrane returned to the original position.

Scale bar, 50 μm.

**Supplemental videos:**

**Video1 Sham CC:** Related to **Fig. 1C.** This is an example video of 3D confocal imaging stack from the corpus callosum (CC) of a sham Thy1-YFP transgenic mouse. In this control video, YFP fluorescence signals are in green and Hoechst (a nuclear dye) in blue.

**Video2 CHIM CC:** Related to **Fig. 1C.** This is an example video of 3D confocal imaging stack from CC of a Thy1-YFP transgenic mouse received CHIMERA impact (0 hour; immediate fixation after impact). YFP fluorescence signals are in green and Hoechst (a nuclear dye) in blue. This video shows that axonal varicosities clearly formed along multiple axons.

**Video3 Sham EC:** Related to **Fig. 1D.** This is an example video of 3D confocal imaging stack from the external capsule (EC) of a sham Thy1-YFP transgenic mouse. In this control video, YFP fluorescence signals are in green and Hoechst (a nuclear dye) in blue.

**Video4 CHIM EC:** Related to **Fig. 1D.** This is an example video of 3D confocal imaging stack from EC of a Thy1-YFP transgenic mouse received CHIMERA impact (0 hour; immediate fixation after impact). YFP fluorescence signals are in green and Hoechst (a nuclear dye) in blue. This video shows that axonal varicosities clearly formed along multiple axons.

**Video5 Sham Ctx:** Related to **Fig. 1E.** This is an example video of 3D confocal imaging stack from the cortex (Ctx) of a sham Thy1-YFP transgenic mouse. In this control video, YFP fluorescence signals are in green and Hoechst (a nuclear dye) in blue. This video shows a main thick axon and several thinner axons nearby without clear varicosities. The mild enlargements along several thin axons are potential presynaptic boutons and not the result of mechanical impact.

**Video6 CHIM Ctx:** Related to **Fig. 1E.** This is an example video of 3D confocal imaging stack from Ctx of a Thy1-YFP transgenic mouse received CHIMERA impact (0 hour; immediate fixation after impact). YFP fluorescence signals are in green and Hoechst (a nuclear dye) in blue. This video shows that axonal varicosities clearly formed not only along a main thick axon, but along some thin axons nearby as well.
